# Self-activating G protein α subunits engage seven-transmembrane regulator of G protein signaling (RGS) proteins and a Rho guanine nucleotide exchange factor effector in the amoeba *Naegleria fowleri*

**DOI:** 10.1016/j.jbc.2022.102167

**Published:** 2022-06-20

**Authors:** Dustin E. Bosch, William R. Jeck, David P. Siderovski

**Affiliations:** 1 Department of Pathology, Roy J. and Lucille A. Carver College of Medicine, University of Iowa, Iowa City, Iowa, USA; 2 Department of Pathology, Duke University School of Medicine, Durham, North Carolina, USA; 3 Department of Pharmacology & Neuroscience, University of North Texas Health Science Center, Fort Worth, Texas, USA

**Keywords:** cell signaling, brain, enzyme kinetics, enzyme structure, G protein, GTPase activating protein, microbiology, protein crystallization, protein structure, signal transduction, AMF, aluminum magnesium and fluoride, GAP, GTPase-accelerating protein, GEF, guanine nucleotide exchange factor, GPCR, G protein–coupled receptor, MSA, multiple sequence alignment, P-loop, phosphate binding loop, PLC, phospholipase C, RGS, regulator of G protein signaling protein, r.m.s.d, root mean square deviation, SPR, surface plasmon resonance

## Abstract

The free-living amoeba *Naegleria fowleri* is a causative agent of primary amoebic meningoencephalitis and is highly resistant to current therapies, resulting in mortality rates >97%. As many therapeutics target G protein–centered signal transduction pathways, further understanding the functional significance of G protein signaling within *N. fowleri* should aid future drug discovery against this pathogen. Here, we report that the *N. fowleri* genome encodes numerous transcribed G protein signaling components, including G protein–coupled receptors, heterotrimeric G protein subunits, regulator of G protein signaling (RGS) proteins, and candidate Gα effector proteins. We found *N. fowleri* Gα subunits have diverse nucleotide cycling kinetics; Nf Gα5 and Gα7 exhibit more rapid nucleotide exchange than GTP hydrolysis (*i.e.*, “self-activating” behavior). A crystal structure of Nf Gα7 highlights the stability of its nucleotide-free state, consistent with its rapid nucleotide exchange. Variations in the phosphate binding loop also contribute to nucleotide cycling differences among Gα subunits. Similar to plant G protein signaling pathways, *N. fowleri* Gα subunits selectively engage members of a large seven-transmembrane RGS protein family, resulting in acceleration of GTP hydrolysis. We show Nf Gα2 and Gα3 directly interact with a candidate Gα effector protein, RGS-RhoGEF, similar to mammalian Gα_12/13_ signaling pathways. We demonstrate Nf Gα2 and Gα3 each engage RGS-RhoGEF through a canonical Gα/RGS domain interface, suggesting a shared evolutionary origin with G protein signaling in the enteric pathogen *Entamoeba histolytica*. These findings further illuminate the evolution of G protein signaling and identify potential targets of pharmacological manipulation in *N. fowleri*.

The free-living amoeba *Naegleria fowleri* is the causative agent of primary amoebic meningoencephalitis, a rare infection with mortality rates >97% in the United States (cdc.gov, (1, 2)). The organism is found primarily in fresh water, as well as soil, and cycles among trophozoite, flagellated, and encysted forms (1, 3, 4). Human infection is established by intranasal exposure, typically during swimming in warm freshwater bodies, although ritual nasal cleansing (ablution) and the use of medical sinonasal rinsing devices have also been implicated (5, 6). *N. fowleri* trophozoites access the cranial cavity by tracking along olfactory neurons and crossing the cribriform plate (1, 7). The amoebae incite a robust and destructive neutrophilic inflammatory response in the meninges and brain, in contrast to the type IV hypersensitivity response elicited in the brain by other free living amoebae such as *Balamuthia* or *Acanthamoeba* (8). The resulting devastating brain injury is thought to result primarily from an amplified immune response, rather than direct toxicity or phagocytosis by the parasite, as implied by the misnomer “brain-eating amoeba” (8). Primary amoebic meningoencephalitis progresses rapidly, leading almost invariably to death within ∼5 days (9). Symptoms of *N. fowleri* infection may mimic the more common etiologies of meningitis (bacterial and viral), complicating diagnosis and potentially delaying therapy (9, 10). A review of confirmed *N. fowleri* cases revealed a myriad of treatment approaches including combinations of antifungal and antiparasitic drugs that unfortunately lack significant impact on survival (<3% in the US) (2). Investigation of potential therapeutic targets is therefore critically needed for this rare but nearly universally fatal disease.

G protein signaling pathway modulators comprise approximately one-fourth of all currently FDA-approved drugs, with the most frequent targets being the seven-transmembrane G protein–coupled receptors (GPCRs) at the top of the pathway (11). GPCRs are specifically activated by a wide variety of extracellular cues such as hormones, neurotransmitters, chemokines, and photons and activate cytoplasmic heterotrimeric G proteins composed of Gα, Gβ, and Gγ subunits (12). Receptor-catalyzed exchange of GDP for GTP on the Gα subunit induces a conformational change dominated by three mobile switch regions (13). The activated Gα⋅GTP separates from the Gβγ heterodimer, both of which engage effector proteins and promote second messenger signaling (12). Signaling is terminated by GTP hydrolysis on the Gα subunit, a reaction accelerated by regulators of G protein signaling (RGS proteins) and leading to the re-formation of the Gαβγ heterotrimer (14, 15). Canonical RGS proteins serve as GTPase-accelerating proteins (GAPs) by stabilizing the switch regions of GTP-bound Gα subunits in the transition state (16). Within the animal kingdom, Gα subunits can be classified into four subfamilies: Gα_s_ and Gα_i/o_ subfamilies stimulate and inhibit adenylyl cyclase, respectively; Gα_q_ family members engage phospholipase Cβ isoforms; and the Gα_12/13_ subfamily activates a family of Rho GTPase guanine nucleotide exchange factors (GEFs) containing RGS-like domains (RGS-RhoGEFs) (17, 18, 19).

In contrast to the animal kingdom, plant, fungal, and protozoan Gα subunits exhibit greater sequence divergence and, correspondingly, diverse interactions with signaling partners (20). For instance, *Saccharomyces cerevisiae* GPA1 and *Arabidopsis thaliana* GPA1 do not engage homologs to mammalian Gα subunit effectors; instead, the Gβγ dimer is thought to play a dominant role promoting downstream signaling in fungi and plants (21, 22). An additional important difference of many plant Gα subunits such as *A. thaliana* GPA1 (23, 24) and some protozoan Gα subunits like those of *Trichomonas vaginalis* (25) is relatively rapid nucleotide exchange activity in the absence of receptor influence. In these Gα subunits, GTP hydrolysis rather than nucleotide exchange is the rate limiting step of the nucleotide cycle, allowing accumulation of the activated Gα⋅GTP species in the cytoplasm, independent of a GPCR or other GEF, referred to elsewhere as “self-activation” (24, 26). Plant Gα subunits are known to engage seven-transmembrane RGS proteins (7TM RGS) that accelerate the rate-limiting GTP hydrolysis step, likely with modulation by extracellular cues, as exemplified by the glucose-responsive *A. thaliana* 7TM RGS protein AtRGS1 (23, 24). Other protozoan Gα subunits exhibit the typical nucleotide cycle pattern of rate-limiting nucleotide exchange and thus presumably require activation by a GEF such as a GPCR (27). One such Gα subunit from the enteric pathogen *Entamoeba histolytica*, EhGα1, engages the Gα effector EhRGS-RhoGEF, leading to Rho family GTPase activation and modulation of pathogenic behaviors such as migration, extracellular matrix invasion, and host cell killing (28, 29). A more extensive array of G protein signaling components, including cyclic AMP receptors typified by cAR1, are utilized by the slime mold *Dictyostelium discoideum* for processes such as chemotaxis, development, and quorum-sensing (30, 31).

The availability of *N. fowleri* genome sequences (32, 33) along with publicly available RNAseq transcriptome data has provided opportunity for the identification and validation of potential therapeutic targets. Of note, there is substantial divergence at the genome sequence level from the nonpathogenic-related species *Naegleria gruberi* (32, 34). In the present study, we identify and characterize G protein signaling components encoded by the *N. fowleri* genome that may be amenable to future pharmacological manipulation.

## Results

### Putative G protein signaling components encoded by the *N. fowleri* genome

**Figure 1:**
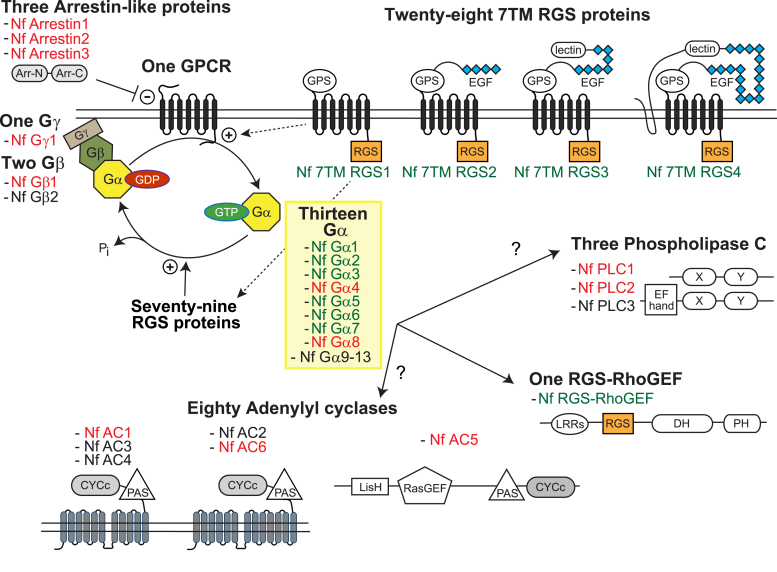
**Candidate heterotrimeric G protein signaling components encoded by the *Naegleria fowleri* genome.** Homologs of known G protein signaling components were identified by hidden Markov model searches of the *N. fowleri* genome (32). *Green text* indicates successful (and *red text* indicates attempted) PCR-based cloning of open reading frames from genomic DNA and expression as recombinant proteins in *E. coli*. Domain abbreviations are as follows: Arr-N and Arr-C, N- and C-terminal arrestin-like domains; GPS, GPCR proteolytic site motif; EGF, epidermal growth factor-like domain; RGS, regulator of G protein signaling domain or “RGS-box”; EF hand, calcium binding motif; X, phospholipase C X catalytic domain; Y, phospholipase C Y catalytic domain; CYCc, adenylyl/guanylyl cyclase catalytic domain; PAS, signal sensor motif; LisH, lissencephaly type 1-like homology motif; RasGEF, Ras GTPase guanine nucleotide exchange factor; LRRs, leucine rich repeats; DH, Dbl homology domain; PH, pleckstrin homology domain.

Heterotrimeric G protein subunits, their nucleotide cycle regulators, and candidate Gα effectors were identified by bioinformatic interrogation of the *N. fowleri* genome (32) using hidden Markov models. Thirteen putative Gα, two Gβ, and one Gγ subunits were identified (Fig. 1), and the majority are apparently expressed in trophozoites (Table S1) as evidenced by publicly available RNAseq data (32, 35). A single expressed gene with low homology (29% identity) to *D. discoideum* cyclic AMP receptor-like proteins (36) was identified as a candidate GPCR (AmoebaDB accession NF0059410). Other predicted seven-transmembrane proteins with some features of GPCRs, despite no significant sequence similarity to known receptors, were identified within the *N. fowleri* proteome using 7TMRmine (*data not shown*) (37). The presence of transcribed arrestin-like genes supports the hypothesis of at least one functional GPCR protein in *N. fowleri*, given that the encoded arrestin-like proteins are predicted to have roles in GPCR desensitization, internalization, and recycling (38, 39). *N. fowleri* also expresses a relatively large family of 28 putative seven-transmembrane proteins with RGS domains at the C terminus (Fig. 1)—a fused protein construction as also seen in plants and some other protists such as *Trichomonas* (20, 25, 40). Probably best characterized is the 7TM RGS protein from *A. thaliana*, AtRGS1, that modulates cellular responses to glucose, in part by accelerating GTPase activity on the “self-activating” Gα subunit AtGPA1 (23, 40, 41, 42). A number of the 7TM RGS proteins in *N. fowleri* harbor GPCR proteolytic site motifs (Fig. 1), reminiscent of the adhesion GPCRs that are activated *via* cell–cell or cell–matrix contact (43). Consistent with this suggested function, several Nf 7TM RGS proteins have complex extracellular N termini with predicted epidermal growth factor–like repeats and lectin domains (*e.g.*, Nf 7TM RGS2, Nf 7TM RGS3, and Nf 7TM RGS4; Fig. 1). In addition to 7TM RGS proteins, the *N. fowleri* genome encodes a large RGS protein family, with 79 additional RGS domain-containing proteins (beyond the 7TM RGS protein class) and a single RGS-RhoGEF protein with a multidomain structure (Fig. 1) similar to the Gα effector in *E. histolytica*, EhRGS-RhoGEF (27, 29), despite low protein sequence similarity (20%). Three phospholipase C (PLC) genes are present within the *N. fowleri* genome, although none encodes sufficient protein sequence similarity with mammalian PLCs to allow subclassification, such as among the PLCβ isozymes that are Gα_q_ effectors in mammals (19). Relatively simplified PLC domain structures with catalytic X-box and Y-box domains, and EF hands suggest calcium regulation (Fig. 1). A remarkably large family of 80 putative adenylyl/guanylyl cyclase proteins containing a catalytic CYCc domain are present in the *N. fowleri* genome (Fig. 1), 62 of which are apparently simultaneously expressed in trophozoites by RNAseq (FPKM > 20th percentile) (32, 35). These putative cyclic nucleotide-forming enzymes exhibit diverse topologies and domain combinations, including predicted cytoplasmic proteins (*e.g.*, Nf AC5) and proteins with variable predicted transmembrane helices (Fig. 1).

### Phylogenetic analyses and nucleotide exchange characteristics of *N. fowleri* Gα subunits

Gα subunits are signaling hubs with distinct downstream effectors that can be predicted in mammals and higher eukaryotes based on sequence similarity (17). Phylogenetic analyses were performed based on multiple sequence alignments (MSAs) of the Gα subunits encoded within the *N. fowleri* genome, as compared to Gα MSAs from humans and select model organisms (Fig. S1). Clear phylogenetic relationships were apparent among subfamilies of Gα subunits from humans, *Dictyostelium rerio*, *Dictyostelium melanogaster*, and *C. elegans*; in contrast, those from protists such as *N. fowleri*, *D. discoideum*, *T. vaginalis*, and *E. histolytica*, from fungi such as *S. cerevisiae*, and from the plants *A. thaliana* and *O. sativa* are more distantly related. Of note, “self-activating” Gα subunits (those with known rapid nucleotide exchange rates, such as *A. thaliana* GPA1 and *T. vaginalis* Gα1 (23, 24, 27)) do not show clear phylogenetic relationships (Fig. S1).

**Figure 2:**
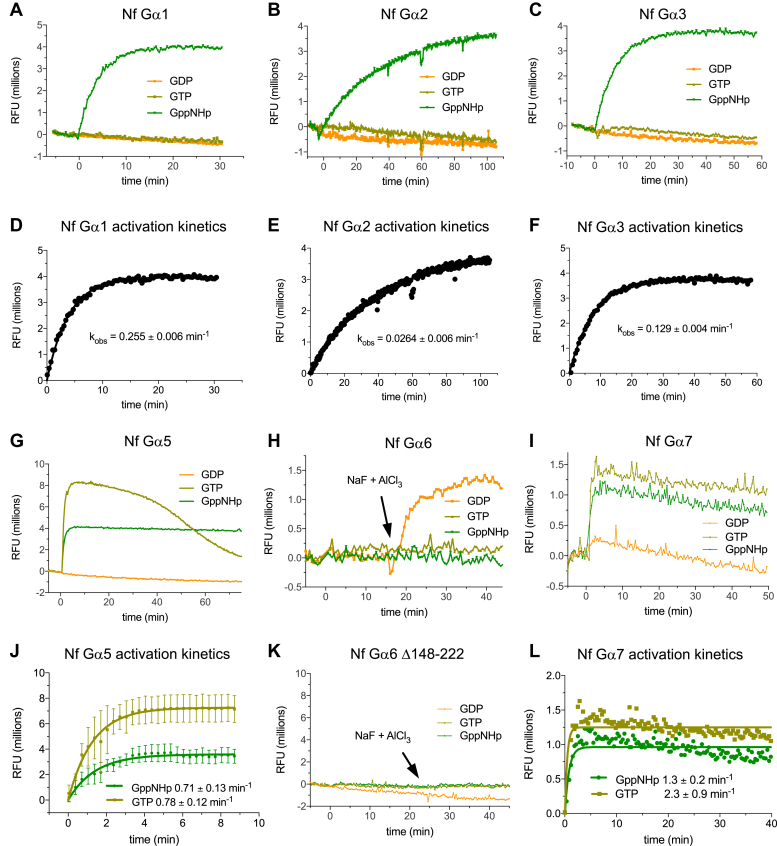
**Nucleotide-dependent activation of *N. fowleri* Gα subunits.** Indicated recombinant purified Gα subunits were mixed with nucleotide at time zero, and the intrinsic tryptophan fluorescence monitored to detect conformational change. Nf Gα1, NfGα2, and Nf Gα3 underwent conformational change detected in the presence of the nonhydrolyzable GTP analog GppNHp (*A*–*C*). Activation rates were obtained from data shown in panels *D*–*F*. Nf Gα5 and Nf Gα7 exhibited “self-activation” (*G* and *I*) and assumed the active conformation in the presence of GTP, indicating that GTP hydrolysis, rather than nucleotide exchange, is rate limiting under these conditions. Activation rates were obtained from data shown in panels *J* and *L*. Nf Gα6 was not activated by guanine nucleotides, but conformational change was detected upon addition of NaF and AlCl_3_ in the presence of magnesium (AMF; *H*). Deletion of a predicted low complexity loop in Nf Gα6 (a.a. 148–222) abolished activation by AMF (*K*). All traces are a single representative from three independent experiments, except panel *J* which reflects mean and standard deviation of three independent experiments. AMF, aluminum magnesium and fluoride; RFU, relative fluorescence units.

To examine the functionality and nucleotide cycling characteristics of Gα subunits in *N. fowleri*, six family members were produced as recombinant proteins from *Entamoeba coli* (Fig. S2). Conformational change upon activation of many Gα subunits can be detected as changes in intrinsic tryptophan fluorescence, primarily effected by a tryptophan on switch 2 (44, 45), a fluorescent residue which is universally conserved among the *N. fowleri* Gα subunits (Fig. S3). When purified from *E. coli*, five *N. fowleri* Gα subunits exhibited the expected increase in tryptophan fluorescence upon nucleotide activation (Fig. 2). Three of these Gα subunits (Nf Gα1–3) exhibited typical activation upon binding to the nonhydrolyzable GTP analog GppNHp (Fig. 2, *A*–*C*), while GTP was insufficient for detectable activation, consistent with nucleotide exchange being the rate limiting step in the nucleotide cycle. Observed kinetics of activation for Nf Gα1–3 ranged over an approximate order of magnitude (∼0.03–0.3 min^−1^; Fig. 2, *D*–*F*). In contrast, Nf Gα5 and Nf Gα7 each achieved the active conformation in the presence of either GppNHp or GTP (Fig. 2, *G* and *I*), consistent with “self-activation” and GTP hydrolysis being the rate limiting step of nucleotide cycling. As observed for other Gα subunits with rapid nucleotide exchange (*e.g.*, ref. (24)), the intrinsic tryptophan fluorescence of Nf Gα5 returned slowly to near-baseline levels after multiple rounds of hydrolysis and exhaustion of available GTP (Fig. 2*G*). Nf Gα5 and Nf Gα7 also exhibit significantly more rapid activation kinetics (0.7 and 1.3 min^−1^) as compared to other Nf Gα subunits tested (Fig. 2, *J* and *L*). Nf Gα6 did not display a significant change in tryptophan fluorescence upon addition of either GTP or GppNHp (Fig. 2*H*). However, activation was achieved by addition of GDP, NaF, and AlCl_3_ in the presence of Mg^2+^ (*i.e.*, forming aluminum magnesium and fluoride [AMF]) to mimic the hydrolysis transition state. This pattern of activation is reminiscent of human Gα_q_, which exhibits negligible nucleotide exchange activity *in vitro* in the absence of a ligand-activated GPCR but is rapidly activated by addition of AMF (46). Nf Gα6 contains a relatively low-complexity polypeptide insertion N terminal to the predicted switch 1 region, as highlighted by MSA (Fig. S3). To examine the potential role of this insertion in modulating nucleotide exchange, a deletion mutant (Δ148–222) was constructed and produced as a recombinant protein from *E. coli* (Fig. S2). However, Nf Gα6^Δ148–222^ was not activated by any nucleotide or AMF (Fig. 2*K*). Two of several possible explanations are that residues 148 to 222 are required for Nf Gα6 to assume the active conformation or that deletion of these residues results in loss of specific activity (*e.g.*, misfolding).

### Structure of a self-activating Gα in the nucleotide-free state

**Figure 3:**
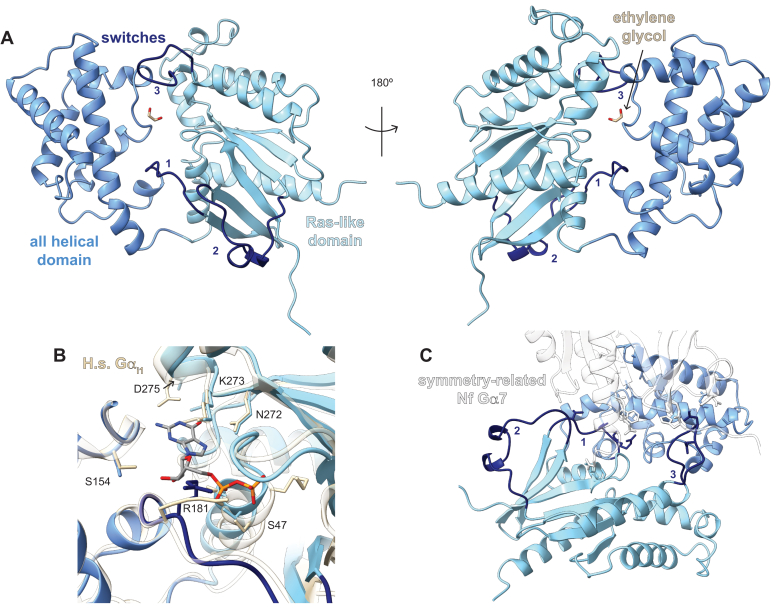
**Structural model of Nf Gα7 in the nucleotide-free state as obtained by X-ray crystallography.***A*, the overall structure of Nf Gα7 exhibited domain architecture and secondary structure highly similar to mammalian, plant, and protozoan Gα subunits despite low protein sequence identity. Although GDP was present in the crystallization conditions, electron density for nucleotide was absent. Switch 2 is extended away from the nucleotide binding site, which is typical of other Gα subunit structures in inactive states. *B*, the overall structure of Nf Gα7 is highly similar to human Gα_i1_ (PDB id 1GIT), and key nucleotide-interacting residues are well conserved. Important conformational differences in the nucleotide-free Nf Gα7 include rotation (∼90°) away from the nucleotide binding site of Asp275, the key residue in the highly conserved guanine binding NKxD motif, and distinct backbone positioning and side chain rotamer of Arg181 partially obstructing the nucleotide binding site. *C*, contacts of the switch regions with the neighboring asymmetric unit may influence their conformation in the structural model.

To better understand the nucleotide cycling characteristics of the “self-activating” *N. fowleri* G proteins, crystallographic studies were attempted on both Nf Gα5 and Nf Gα7. A structural model based on diffraction data (1.7 Å resolution) was obtained for the self-activating Nf Gα7 crystallized in the presence of GDP (Fig. 3; PDB id 6NE6; ref. (47)). The structural data resulting from collaborative efforts with the Seattle Structural Genomics Center for Infectious Disease were made publicly available in 2019 and briefly mentioned in a prior Seattle Structural Genomics Center for Infectious Disease (SSGCID) publication (47). However, depictions, comparisons, and analyses in the current work have not been published elsewhere. The overall structure was highly similar to mammalian Gα subunits such as Gα_i1_ (PDB id 1GIT, DALI server z score 34, Cα r.m.s.d. 2.2 Å, protein sequence identity 37%), the protozoan EhGα1 from *E. histolytica* (PDB id 4FID, z score 30, Cα r.m.s.d. 2.3 Å, protein sequence identity 31%), and the plant AtGPA1 from *A. thaliana* (PDB id 2XTZ, z score 25, Cα r.m.s.d. 2.3 Å, protein sequence identity 34%) (48). Among the three switch regions that dominate nucleotide-dependent conformational changes in other Gα structures and their interactions with effectors, Nf Gα7 switch 2 (a.a. 204–222) is modeled in a position that is extended away from the nucleotide binding site, similar to previous structural models of other Gα subunits in the inactive (GDP-bound) state (Fig. 3).

Within the electron density data, no nucleotide was observable in the catalytic site of Nf Gα7, which was seen instead to be occupied by solvent and an ordered ethylene glycol, a chemical present in the cryoprotectant solution (Fig. 3). To our knowledge, this is the first crystallographic snapshot of an isolated nucleotide-free Gα subunit, although GPCR/G protein heterotrimer complex structures have also lacked nucleotide (49, 50). The marked shift in the spatial relationship between the all helical and Ras-like domains and the shift of the α5 helix observed in GPCR/G protein complex structures is absent in nucleotide-free Nf Gα7. However, the possibility of similar conformational changes in solution cannot be excluded based on this crystallographic snapshot.

A comparison of the Nf Gα7 nucleotide binding site with the structurally similar GDP-bound human Gα_i1_ (PDB id 1GIT) revealed highly conserved nucleotide-interacting residues (Fig. 3*B*). Asp275 of the NKxD motif stringently conserved across GTPases (51) was rotated away from the nucleotide binding site (D275 in light blue within Fig. 3*B*); furthermore, Arg181 of Nf Gα7, a conserved switch 1 residue required for efficient GTP hydrolysis (52), adopted a side-chain rotamer that partially obstructs the nucleotide binding site (R181 in *dark blue* in Fig. 3*B*). However, the conformations of switch region residues within Nf Gα7, including Arg181, may be influenced by crystallographic contacts observed with the neighboring asymmetric unit (Fig. 3*C*). Previous structural and molecular dynamics studies of the “self-activating” GPA1 from *A. thaliana* have suggested that heightened mobility of the all-helical domain, reflected as high B factors in the crystal structure, serves as a principal mechanism of rapid nucleotide exchange (53, 54). In contrast, the structural model of Nf Gα7 has no significant average B factor differences between all helical and Ras-like domains.

### *N. fowleri* RGS proteins accelerate Gα GTP hydrolysis

**Figure 4:**
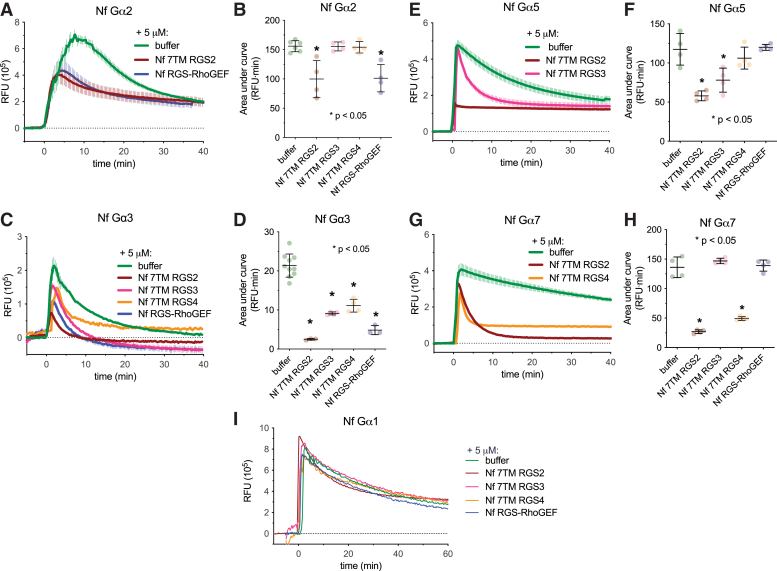
**An RGS-RhoGEF effector and 7TM RGS proteins are selective GTPase accelerating proteins for *N. fowleri* Gα subunits.** Four recombinant *N. fowleri* RGS domains (at 5 μM concentration) were tested consecutively against five Gα subunits for GTPase accelerating protein (GAP) activity using a fluorescent nucleotide substrate (55). *A*, Nf Gα2 showed accelerated GTP hydrolysis in the present of Nf 7TM RGS2 and Nf RGS-RhoGEF. A significant reduction in area under the curve (AUC) indicates GAP activity (*B*, *D*, *F* and *H*). *E*, Nf 7TM RGS2 and Nf 7TM RGS3 had GAP activity on Nf Gα5. *C*, Nf Gα3 showed accelerated GTPase activity in the presence of all RGS domains tested, while hydrolysis on Nf Gα1 (*I*) was unaffected by each. *G*, Nf 7TM RGS2 and Nf 7TM RGS4 had GAP activity on Nf Gα7. Time course and AUC error bars reflect standard deviation for independent experiments (n = 3–10). 7TM RGS, seven-transmembrane RGS proteins; RGS, RGS, regulator of G protein signaling domain.

To identify potential transmembrane interaction partners and downstream effectors for Nf Gα7 and other Nf Gα subunits, the isolated RGS domains from four 7TM RGS proteins (named Nf 7TM RGS1–4; Fig. 1) and Nf RGS-RhoGEF were produced as recombinant proteins from *E. coli* (Fig. S2). Each Gα/RGS domain combination was screened for a functional interaction using a previously described fluorescent nucleotide (BODIPYFL-GTP) hydrolysis assay for GAP activity (55). The RGS domain of Nf 7TM RGS1 interfered with baseline fluorescence of BODIPYFL-GTP, rendering uninterpretable fluorescence time courses; it was therefore excluded from further study. Similarly, Nf Gα6 was excluded because no appreciable nucleotide binding or hydrolysis was detected by incubation with BODIPYFL-GTP, consistent with the inability to activate Nf Gα6 with GTP or GppNHp in intrinsic tryptophan fluorescence assays (Fig. 2*H*). Each of the remaining four RGS domains were mixed with Nf Gα1–3, Nf Gα5, or Nf Gα7, and GAP activity by these RGS domains subsequently detected as a significant decrease in area under the fluorescence time course curve (Fig. 4) (55). No functional interaction of Nf Gα1 was observed with RGS domains, while both Nf 7TM RGS2 and Nf RGS-RhoGEF served as GAPs for Nf Gα2 (Fig. 4, *A*, *B* and *I*). Each of the RGS domains exhibited significant GAP activity on Nf Gα3, suggesting promiscuous coupling of this particular Nf Gα subunit to RGS proteins (Fig. 4, *C* and *D*). Nf 7TM RGS2 and Nf 7TM RGS3 served as GAPs for Nf Gα5, while Nf Gα7 engaged Nf 7TM RGS2 and Nf 7TM RGS4 (Fig. 4, *E*–*H*).

**Figure 5:**
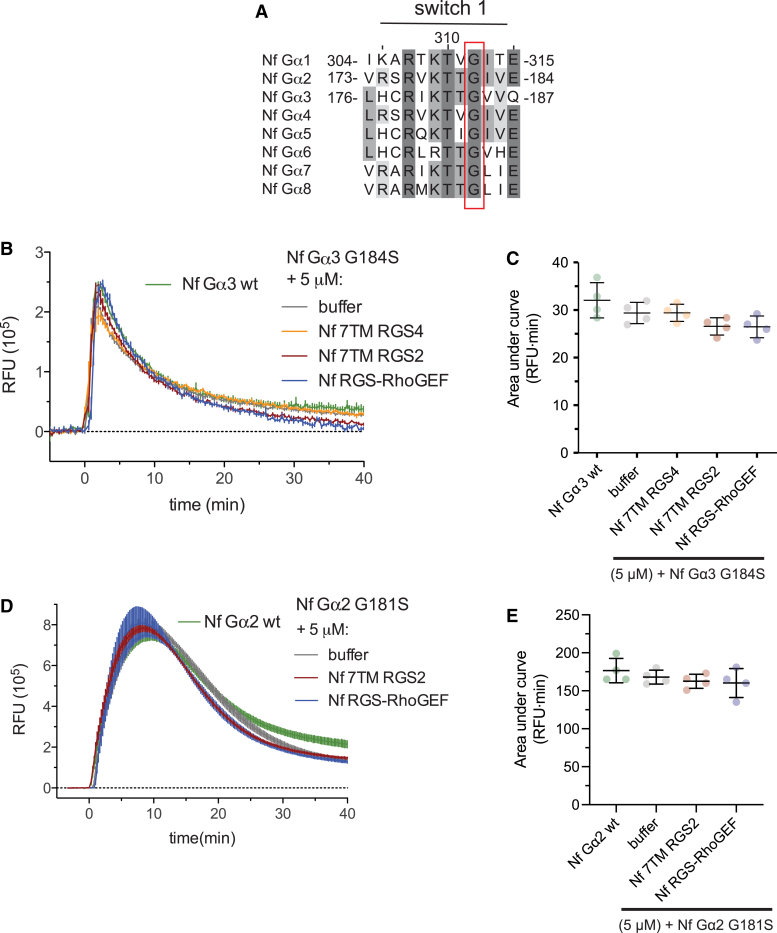
**A switch 1 RGS insensitivity mutant eliminates GAP activity and demonstrates canonical RGS/Gα interactions.***A*, a highly conserved glycine residue in switch 1, when mutated to serine (the “RGS insensitivity” mutation; ref. (57)) eliminates GAP activity of canonical Gα/RGS pairs. *B* and *C*, GTP hydrolysis on Nf Gα3 G184S is unaffected by presence of 7TM RGS domains and the Nf RGS-RhoGEF putative effector. *D* and *E*, similarly, no RGS domain–mediated GAP activity was observed on Nf Gα2 G181S. Time course and AUC error bars reflect standard deviation for independent experiments (n = 4). 7TM RGS, seven-transmembrane RGS proteins; GAP, GTPase-accelerating proteins; RGS, RGS, regulator of G protein signaling domain.

A subtle mutation of a conserved Gα switch 1 glycine to serine, known as the “RGS insensitivity” mutation (56, 57), disrupts interactions of canonical RGS domains with their Gα subunit partners, while mammalian RGS-RhoGEF “RGS-like” domain/Gα interactions are not affected by this G-to-S mutation. The switch 1 glycine is conserved across *N. fowleri* Gα subunits (Fig. 5*A*), and mutation of this position to serine in Nf Gα2(G181S) and NfGα3(G184S) disrupted GAP activity by all tested RGS domains (Fig. 5, *B*–*E*). Of note, interaction of both NfGα2 and NfGα3 with the RGS domain of Nf RGS-RhoGEF was disrupted by the RGS insensitivity mutation, indicating canonical RGS domain/Gα interactions rather than an interface akin to mammalian RGS-RhoGEF/Gα pairs. This mode of Gα and effector interaction (*i.e.*, using a canonical RGS domain rather than an “RGS-like” or “RGS-homology” domain) was previously observed in *E. histolytica*, suggesting a shared evolutionary origin (29).

**Figure 6:**
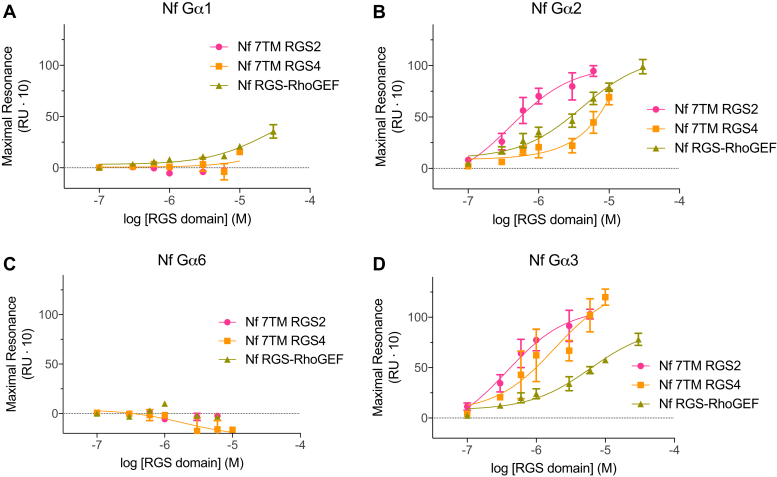
**Nf Gα2 and Nf Gα3 directly engage both 7TM RGS proteins and Nf RGS-RhoGEF.** Surface plasmon resonance was used to quantify the affinity of interaction among four recombinant *N. fowleri* Gα subunits (immobilized) and three RGS domains (analyte). *A*, Nf Gα1 showed low affinity interaction with Nf RGS-RhoGEF RGS domain. *B* and *D*, Nf Gα2 and Nf Gα3 exhibited binding with all three RGS domains, although the order of interaction affinities differed. *C*, no appreciable interactions with RGS domains were detected for Gα6. All interactions were highly selective for the transition state mimetic AMF-bound form of Gα (Figs. S4–S6). Semilogarithmic binding curves and dissociation constants are shown for AMF states only. Error bars are representative of triplicate injections (n = 3) in one representative experiment. 7TM RGS, seven-transmembrane RGS proteins; AMF, aluminum magnesium and fluoride; RGS, RGS, regulator of G protein signaling domain.

Direct binding interactions between selected *N. fowleri* Gα subunits and purified recombinant RGS domains were also examined by surface plasmon resonance (SPR), and binding affinities quantified (Fig. 6; additional data in Figs. S4–S6). All observed Gα/RGS interactions were selective for the transition state mimetic (GDP and AlF_4_^−^ bound) form of Gα, consistent with prior studies of RGS domain binding selectivity (*e.g.*, ref. (29, 58)) (Figs. S4–S6). No significant binding to RGS domains within physiologically relevant concentration ranges was detected for either Nf Gα6 or Nf Gα1 (Fig. 6, *A* and *C*), consistent with a lack of measurable GAP activity on these two Gα subunits (*e.g.*, Fig. 4*I*). Nf Gα2 exhibited preferential binding to the RGS domain of Nf 7TM RGS2 (K_D_ = 630 ± 190 nM) and lower affinity interaction with Nf RGS-RhoGEF (K_D_ = 2.4 ± 0.5 μM). Increased resonance of the Nf Gα2 surface with high concentrations of Nf 7TM RGS4 likely represents nonspecific binding, as indicated by the atypical, approximately linear binding curve (Fig. S5). In support of this hypothesis, no significant GAP activity was observed for this Gα/RGS domain pair at 5 μM RGS protein concentration (Fig. 4*B*). Nf Gα3 exhibited specific binding with three RGS domains: Nf 7TM RGS2 (K_D_ = 550 ± 160 nM), Nf 7TM RGS4 (K_D_ = 1.8 ± 0.5 μM), and Nf RGS-RhoGEF (K_D_ = 3.3 ± 0.8 μM) (Fig. 6). No binding of Nf Gα3 to Nf 7TM RGS3 was detected in any nucleotide state (*data not shown*). Although significant reduction of AUC was observed for this pair in BODIPYFL-GTP hydrolysis assays (Fig. 4*D*), the buffer and Nf 7TM RGS3 fluorescence time courses exhibit strikingly similar shapes (Fig. 4*C*), and there is substantial deflection of the Nf 7TM RGS3 curve below baseline, suggesting a nonspecific fluorescent effect, rather than true acceleration of GTP hydrolysis, with this particular assay pairing.

### Phosphate binding loop variation among *N. fowleri* Gα subunits contributes to nucleotide cycling characteristics and RGS domain interactions

**Figure 7:**
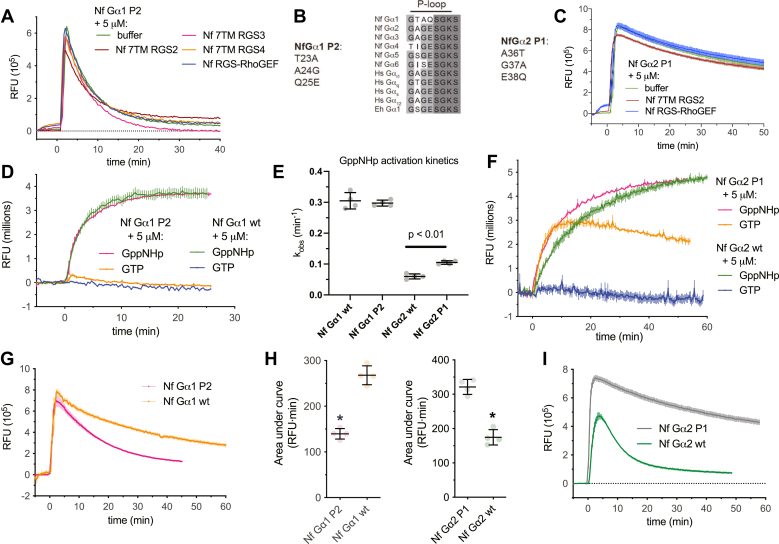
**The unique P-loop of Nf Gα1 confers relatively slow GTPase kinetics and contributes to RGS domain selectivity.** Protein sequence alignment of *N. fowleri* Gα subunits revealed three unique Nf Gα1 residues (a.a. 23–25) within the otherwise highly conserved phosphate binding loop (P-loop) (*B*). Mutation of these three amino acids to the corresponding residues from Nf Gα2 (36, 37, 38) had no significant effect on activation kinetics as measured by intrinsic tryptophan fluorescence (*D* and *E*) but significantly increased the efficiency of GTP hydrolysis (*G* and *H*; *p* < 0.01). The GTPase activity of the Nf Gα1 P2 chimera also was not affected by RGS domains (*A*). In the converse set of experiments replacing Nf Gα2 residues 36 to 38 with the corresponding Nf Gα1 amino acids 23 to 25, the chimeric protein exhibited a significantly faster rate of activation by GppNHp (*E* and *F*; *p* < 0.01) and assumed an active conformation in the presence of GTP. The efficiency of GTP hydrolysis was significantly reduced in Nf Gα2 P1 (*I* and *H*; ∗ indicates *p* < 0.01). In contrast to wildtype Nf Gα2 (Fig. 4), the GTPase activity of Nf Gα2 P1 was not accelerated by RGS domains from Nf 7TM RGS2 or Nf RGS-RhoGEF (*C*). Time course data represent mean and standard deviation of independent experiments (n = 3–4). Kinetic values and areas under curve are shown as mean with standard deviation (n = 4). 7TM RGS, seven-transmembrane RGS proteins; RGS, RGS, regulator of G protein signaling domain.

The phosphate binding loop (P-loop) is a highly conserved motif among G proteins, as well as within ATP-binding kinases (59). The P-loop is intimately associated with the bound nucleotide in Gα subunits; as such, mutations in this region are known to reduce nucleotide hydrolysis or impair activation (45, 60). A MSA of selected *N. fowleri* Gα subunits with human and other protist Gα subunits revealed a high degree of overall conservation (Fig. 7*B*). However, the Nf Gα1 sequence deviates significantly in positions 23 to 25, suggesting a role for this region in modulating nucleotide cycling. The Nf Gα1 alanine 24 corresponds to a position with high conservation of glycine among heterotrimeric G proteins and Ras superfamily GTPases, interacting directly with the γ-phosphoryl group of GTP. Missense mutations at this locus to essentially any other residue disrupt GTPase activity in Ras GTPases, rendering them constitutively active, as commonly seen in the oncogenic Ras G12V mutation (61). Mutation of this residue in heterotrimeric G proteins has variable effects on nucleotide cycling. For example, human Gα_i1_ G42V is GTPase deficient and constitutively active, while Gα_i1_ G42R is unable to transition to the active state conformation (45, 60). The position corresponding to Nf Gα1 glutamine 25 is well conserved as a negatively charged residue (typically glutamate) that interacts with bound nucleotide and exhibits interactions with the conserved “arginine finger” (*e.g.*, R181 in Nf Gα7, Fig. 3*B*), implicated in control of nucleotide exchange (62).

To test this hypothesis, P-loop residues 23 to 25 of Nf Gα1 were substituted for the corresponding residues 36 to 38 of Nf Gα2 (“Nf Gα2 P1” chimera), and the converse substitution was also generated to create the “Nf Gα1 P2” chimera (Fig. 7). Wildtype Nf Gα1 and the Nf Gα1 P2 chimera were each activated by GppNHp with indistinguishable kinetics (Fig. 7, *D* and *E*), suggesting similar rates of nucleotide exchange. However, Nf Gα1 P2 exhibited more rapid GTP hydrolysis than wildtype protein (*e.g.*, Fig. 7, *G* and *H*; also compare buffer-only conditions of Fig. 4*I* with Fig. 7*A*). Like wildtype Nf Gα1 (Fig. 4*I*), the Nf Gα1 P2 chimera was not a substrate for any RGS domains examined (Fig. 7*A*). In contrast, the Nf Gα2 P1 chimera displayed impaired GTP hydrolysis compared to wildtype (Fig. 7, *H* and *I*). The Nf Gα2 P1 chimera also exhibited significantly more rapid activation by GppNHp (Fig. 7*E*; 0.10 ± 0.01 min^−1^ compared to 0.058 ± 0.002 min^−1^) and assumed an activated conformation in the presence of GTP (Fig. 7*F*). Unlike wildtype Nf Gα2 (Fig. 4, *A* and *B*), Nf Gα2 P1 did not functionally engage Nf 7TM RGS2 or Nf RGS-RhoGEF in GAP activity assay (Fig. 7*C*). Together these findings indicate that the unusual P-loop of Nf Gα1 (23-TAQ-25) confers a relatively slow GTP hydrolysis rate to the Gα subunit possessing it and likely also contributes to selective engagement of RGS domains.

## Discussion

**Figure 8:**
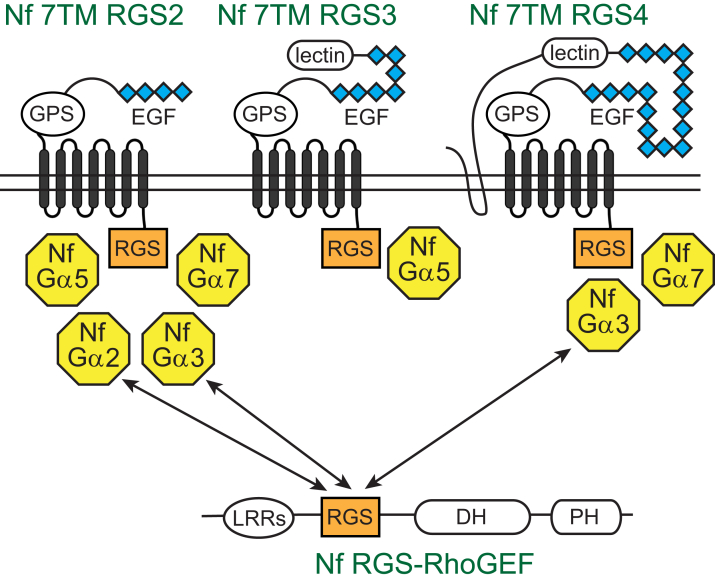
**Summary of selective Gα/RGS domain interactions in *Naegleria fowleri*.** Three 7TM RGS proteins examined in this study selectively engage four Gα subunits, including the “self-activating” subunits Nf Gα5 and Nf Gα7. Nf Gα2 and Nf Gα3 also engage the putative effector Nf RGS-RhoGEF. Each *double arrow* drawn represents an interaction with the Nf RGS-RhoGEF putative effector confirmed by significant GAP activity (Fig. 4) and in the cases of Nf Gα2 and Nf Gα3, nucleotide state-selective binding (Fig. 6). Gα subunit interactions with 7TM RGS domains and corresponding GAP activity are indicated by proximity. GAP, GTPase-accelerating protein; RGS-RhoGEFs, Rho GTPase guanine nucleotide exchange factors containing RGS-like domains.

The *N. fowleri* genome encodes a relatively complex set of heterotrimeric G protein signaling components, many of which are apparently simultaneously expressed in single-celled trophozoites. Given the amenability of G protein signaling to pharmacologic manipulation (11, 15), these pathways provide ample opportunity for the development of chemical probes and (potentially) therapeutics. Of particular interest are the candidate GPCRs in the *N. fowleri* genome; homologs in other organisms respond to extracellular cues including, but not limited to, small molecules (11, 43, 50). Although one candidate *N. fowleri* GPCR (AmoebaDB accession NF0059410) exhibits some similarity to the *D. discoideum* cAR family cyclic AMP receptors with known importance for functions such as chemotaxis and quorum sensing (63); the low overall sequence identity of the *N. fowleri* homolog (29%) limits speculation about potential ligands. The 7TM RGS proteins may provide a similar mode of transmembrane signal transduction, as previously discovered for the plant 7TM RGS protein AtRGS1 that regulates heterotrimeric G protein signaling in response to glucose (64, 65). The remarkable diversity of 28 different 7TM RGS proteins encoded by the *N. fowleri* genome suggests adaptive radiation of this particular signaling modality for detecting extracellular cues. The substantial overlap of RGS domain/Gα subunit interaction specificity in the present study of four Nf 7TM RGS proteins (summarized in Fig. 8) suggests a level of redundancy of downstream signaling for these proteins, should they be responsive to extracellular/environmental agonist cues. A sizable number of 7TM RGS proteins in *N. fowleri*, including Nf 7TM RGS1–4 as illustrated in Figure 1, exhibit extracellular domain structures with epidermal growth factor–like repeats, putative carbohydrate-binding domains, and GPCR proteolytic site motifs reminiscent of the adhesion GPCRs that respond to cell–cell or cell–matrix interactions (43). Whether these (and/or other) 7TM RGS proteins in *N. fowleri* function as cell surface receptors and how extracellular cues may alter GAP activity remains to be determined.

The presence of both “self-activating” Gα subunits rate-limited by GTP hydrolysis (*e.g.*, Nf Gα7) and conventional Gα subunits rate-limited by nucleotide exchange (*e.g.*, as presumably catalyzed by ligand-activated GPCR GEF activity) is unique to *Naegleria* among biochemically characterized species to date (20). A previously conducted evolutionary analysis of heterotrimeric G protein signaling led to a hypothesis that GPCR-mediated activation of slow exchanging Gα subunits arose relatively late in evolutionary history among unikonts, which include animals, fungi, and amoebazoans such as *Dictyostelium* (20). In contrast, the more primitive system of “self-activating” Gα subunits coupling to 7TM and other RGS proteins is dominant among the bikonts, which include Excavata such as *Trichomonas* and *Naegleria*. Indeed, biochemical testing of this division has held true, including demonstrations of the “self-activating” properties of four *Trichomonas* Gα subunits that interact with 7TM RGS proteins (20, 25). However, the mixture of Gα subunits with either slow or fast nucleotide exchange in *Naegleria*, and both types functionally interacting with 7TM RGS proteins (Fig. 8), suggest an earlier evolutionary origin of exchange factor–dependent G protein signaling. The Excavata supergroup containing *Naegleria* (one of six total supergroups) is a highly diverse group of organisms, considered to be of closest relationship to the ancestor of all eukaryotes (66). Although an early horizontal gene transfer event cannot be entirely excluded, our data here indicate the presence of both “self-activating” Gα/7TM RGS signaling and exchange-factor dependent G protein signaling in early eukaryotic evolution.

At this time, the specific biological functions of heterotrimeric G protein signaling pathways in *Naegleria* species remain unknown. However, the nucleotide state–dependent interaction of Nf Gα2 and Nf Gα3 with an RGS-RhoGEF candidate effector suggests crosstalk between at least some aspects of *Naegleria* heterotrimeric G protein signaling and Rho family GTPase signaling, the latter which is conserved across species for regulation of the actin cytoskeleton, transcription, and cell division (67, 68, 69, 70). The domain structure of Nf RGS-RhoGEF containing a canonical RGS domain (not an “RGS-like” domain) and a DH-PH tandem that mirrors the domain organization of the RGS-RhoGEF from *E. histolytica*, although the *Naegleria* protein also contains putative leucine-rich repeats at its N terminus (29, 71). Eh RGS-RhoGEF activates Rho family GTPases downstream of Eh Gα1, which modulates trophozoites behaviors such as migration, invasion, and host cell killing that are dependent on a highly dynamic actin cytoskeleton (29, 72, 73). In contrast with the mammalian RGS-like domain-containing RGS-RhoGEFs that serve as Gα_12/13_ effectors, *Naegleria* and *Entamoeba* RGS-RhoGEF proteins interact with Gα subunits though a canonical RGS/Gα interface, a difference that can be distinguished by the RGS insensitivity mutation on switch 1 (Fig. 5) (29). This finding supports a shared evolutionary origin for Gα interactions with RGS-RhoGEFs in *Naegleria* and *Entamoeba*, likely separate from Gα_12/13_ signaling in the animal kingdom.

## Experimental procedures

### Identification and comparison of putative G protein signaling components

The *N. fowleri* genome sequence (32, 33) was obtained through publicly available databases (NCBI). Open-reading frames were predicted using Augustus (74). Candidate G proteins, RGS proteins, arrestins, and G protein effectors were identified using hidden Markov models (HMMer 3.0, (75)) generated from MSAs (Clustal Omega, (76)) of mammalian homologs. Publicly available RNAseq data (NCBI, (32)) was aligned to the *N. fowleri* reference genome sequence and candidate open reading frames using TopHat 2.1.0 and Cufflinks 2.2.0 (77). Read counts and percent sequence coverage were calculated for select loci to determine relative expression levels. Additional candidate G protein signaling components were identified and expression levels assessed by BLAST searching (78) of *N. fowleri* RNAseq data available through AmoebaDB (amoebadb.org, (35)). The *N. fowleri* Gα subunits and RGS proteins were aligned using T-coffee (79), and BLOSUM62-based neighbor-joining dendrograms derived using Jalview 2.10 (80). Phylogenetic analysis of selected Gα subunits was carried out with Phylogeny.fr (81).

### Cloning of G protein signaling components

Heterotrimeric G protein subunits and isolated RGS domains from candidate RGS proteins were cloned by PCR amplification from genomic DNA of the *N. fowleri* Carter strain (ATCC) and inserted into modified pET-15b *E. coli* expression vectors (pET-His-LIC, *e.g.*, (28)) using ligation-independent cloning to form N-terminal tobacco etch virus protease-cleavable, hexahistidine-tagged fusions, as previously described (69). The predicted flexible N-terminal helices (∼25–35 amino acids) of all Gα subunits were deleted prior to cloning. The putative G protein γ subunit, NfGγ1, was not found within the AmoebaDB RNAseq data; it was cloned *de novo* from *N. fowleri* genomic DNA with sequence 5′-ATGAATAAAATGGCAAACCGTATGAACGACTTTGTGTTGCAACAATTATTGGCAGAAAATCAACGTTTAAGAGAAAGTTTAGAAAGTTGTCGAAAGGCCATCCCAATTTCTGAAGCATGTCGAACTCTAATTGATTATTGCAATGATCACAAGTCGAAGGATATGCTCGTGATGGGAGACCCAACCAATCCATACTGGAATCCACCAAAGGATGGCGGTTGTTGTACCATCATGTAA-3′. Primer sequences, AmoebaDB gene identifiers, and fragments used for biochemical experiments are detailed in Table S1. Introns were removed, and mutations generated using an overlap extension PCR method (82).

### Protein purification

*N. fowleri* Gα subunits were expressed and purified from *E. coli*, essentially as we have previously described for *E. histolytica* EhGα1 (28). For hexahistidine-tagged Gα subunits and RGS domains, transformed B834 *E. coli* were grown to an *A*_600nm_ of 0.7 to 0.8 at 37 °C before induction with 500 μM isopropyl-β-D-thiogalactopyranoside for 14 to 16 h at 20 °C. Cell pellets were resuspended in N1 buffer (for Gα subunits: 50 mM Tris-HCl, pH 8.0, 300 mM NaCl, 10 mM MgCl_2_, 10 mM NaF, 30 μM AlCl_3_, 50 μM GDP, 30 mM imidazole, 5% [w/v] glycerol; for RGS domains: 50 mM Hepes pH 8.0, 150 mM NaCl, 30 mM imidazole, 5% [w/v] glycerol) and lysed at 10,000 kPa using an Avestin Emulsiflex. Cleared lysates were applied to nickel–nitrilotriacetic acid resin (GE Healthcare), washed, and eluted with N1 buffer containing 300 mM imidazole. Eluted protein was resolved using a calibrated size exclusion column (GE Healthcare) with S200 buffer for Gα subunits (50 mM Tris-HCl pH 8.0, 250 mM NaCl, 5 mM DTT, 5% [w/v] glycerol, and 50 μM GDP) or RGS domains (50 mM Tris-HCl pH 8.0, 250 mM NaCl, 5 mM DTT, 5% [w/v] glycerol). Recombinant proteins were analyzed by SDS-PAGE electrophoresis, concentrated to 0.5 to 1.5 mM, and snap frozen in a dry ice and ethanol bath prior to long-term cryostorage.

### Crystallization and structure determination

Crystallization and structure determination were accomplished in collaboration with the SSGCID (47). DEB initiated collaboration toward *Naegleria* G protein signaling component structures with SSGCID and provided plasmids, protein purification methods, and preliminary biochemical data. Hexahistidine-tagged Nf Gα7 in crystallization buffer (25 mM Hepes pH 7.5, 500 mM NaCl, 5% glycerol, 2 mM DTT, 0.025% sodium azide, 10 mM MgCl_2_, 10 mM NaF, 30 μM AlCl_3_, 5 μM GDP) was mixed 1:1 with crystallization solution (16% [w/v] PEG-800, 40 mM potassium phosphate monobasic, 20% [v/v] glycerol). The resulting crystals were cryoprotected with 20% ethylene glycol. Diffraction data collection from a single crystal was performed at the Advanced Photon Source (beamline 21-ID-F), data reduced with XDS, and model refined with Phenix (83). The structural model was refined to a 1.7 Å resolution, with R_work_ 0.161 and R_free_ 0.195 (PDB id 6NE6). Crystallographic data collection and refinement statistics have been described in the previous publication (47).

### Intrinsic tryptophan fluorescence measurements

The key tryptophan residue allowing detection of activation (44) was located within the switch 2 regions of *N. fowleri* Gα subunits 1 through 8 (Fig. S3). Tryptophan fluorescence (excitation and emission wavelengths 284 and 340 nm, respectively) was measured at 20 °C in exchange buffer (100 mM Tris pH 7.5, 100 mM NaCl, 1 mM EDTA, 10 mM MgCl_2_, and 5% glycerol) using a FluoroLog modular spectrofluorometer (Horiba) (28). Recombinant purified *N. fowleri* Gα subunit was added to 500 nM concentration, and a baseline fluorescence established. Guanine nucleotide (1 μM) was then added, and fluorescence monitored at 20 to 30 s intervals. For Gα subunits without measurable activation by a nonhydrolyzable GTP analog (GppNHp), 20 mM NaF and 50 μM AlCl_3_ were added to reaction mixtures containing GDP to assemble the transition-state mimetic form (*i.e.*, GDP-aluminum tetrafluoride). Activation rate constants (k_obs_) were estimated by modeling observed fluorescence using one-phase association in GraphPad Prism 7. All experiments were performed in at least biological triplicate (three or more independent experiments).

### Fluorescent GTP hydrolysis and acceleration by RGS proteins

Fluorescent detection of GTP binding and hydrolysis was conducted essentially as described previously (55). Fluorescence measurements (excitation 485 nm and emission 530 nm) were made at a constant temperature of 20 °C and 30 s intervals using a FluoroLog modular spectrofluorometer (Horiba). All experiments were conducted with constant stirring by magnetic stir bars. Experiments were conducted in TEM buffer (20 mM Tris pH 8.0, 1 mM EDTA, 10 mM MgCl_2_). Recombinant purified RGS protein (5 μM) or an equivalent volume of buffer was diluted in TEM buffer. 100 nM nucleotide, BODIPYFL-GTP (ThermoFisher) was added and allowed to equilibrate for at least 10 min, with stabilization of the fluorescent signal. Baseline fluorescence was indistinguishable across experiments, indicating no effect of RGS proteins on nucleotide fluorescence, with the exception of 7TM RGS1 (excluded from further study). After equilibration, recombinant *N. fowleri* Gα subunits were added to a final concentration of 200 nM. Fluorescence time courses were monitored over 40 to 60 min. Relative fluorescence units were derived by subtraction of baseline fluorescence in the absence of Gα subunit. Area under the curve (AUC) was calculated using GraphPad Prism 7. Reduction in AUC was interpreted as reduced time of the Gα subunit in the GTP-bound state, consistent with GTPase activity acceleration (GAP), as previously described (55). All experiments were conducted with biological replicates (3–10 independent experiments). Statistical significance was defined as *p* < 0.05 using a two-tailed *t* test in GraphPad Prism 7.

### SPR binding measurements

SPR-based measurements of protein–protein interaction were performed on a Proteon XPR36 (Bio-Rad) at the UNC Macromolecular Interactions Facility, essentially as described previously (29). Approximately 5000 resonance units of purified hexahistidine-tagged *N. fowleri* Gα subunits were separately immobilized on a nickel-NTA biosensor chip (Bio-Rad) using covalent capture coupling as previously described (84). Two surfaces with irrelevant proteins, one denatured by injection of sodium hydroxide, served as negative controls. Experiments were performed in running buffer containing 50 mM Hepes (pH 7.4), 150 mM NaCl, 0.05% NP-40 alternative (Calbiochem), 50 μM EDTA, and 1 mM MgCl_2_. Three nucleotide states of the Gα subunits were generated by addition of GDP (100 μM), GppNHp (100 μM), or AMF (100 μM GDP, 20 mM NaF, and 30 μM AlCl_3_) to the running buffer, respectively, and then equilibration with this addition over 2 h. Increasing concentrations of RGS proteins were separately injected at 20 μl/min. Equilibrium affinity constants (K_D_) and kinetic parameters of binding (*k*_a_ [association constant] and *k*_d_ [dissociation constant]) were derived using Proteon Manager software (Bio-Rad) and GraphPad Prism 7. All experiments were conducted with three analyte injections (technical replicates) and performed at least twice on separate surfaces.

## Data availability

All data are contained within the manuscript, except genomic and transcriptomic data which are publicly available at amoebadb.org. Structure coordinates and structure factors were deposited in 2019 and available in the PDB (accession 6NE6).

## Supporting information

This article contains supporting information.

## Conflict of interest

The authors declare that they have no conflicts of interest with the contents of this article.

## Supporting information

Supplemental Table S1 Cloning and recombinant production of heterotrimeric G protein signaling components expressed in *N. fowleri*. G protein signaling components identified by open reading frame prediction and RNAseq data analysis (32) were compared with the partially annotated AmoebaDB database (amoebadb.org, (35)). The associated quantitation of relative expression, fragments per kilobase of transcript per million mapped reads (FPKM) is shown, as well as primer sequences used to attempt PCR amplification from genomic DNA.

Supplemental Figure S1 **Phylogenetic analysis of *N. fowleri* Gα subunits.** Phylogenetic analysis of Gα subunits from *N. fowleri*, humans, and select model organisms was performed with bootstrapping based on a multiple sequence alignment. Branch lengths are represented to scale, and branch points are labeled with bootstrap values. **Supplemental Figure S2**. **Isolation of recombinant *N. fowleri* proteins produced in *E. coli*.** A Coomassie brilliant blue stained gel demonstrates enriched recombinant proteins achieved by a single nickel-NTA affinity step and gel filtration chromatography. ∗ indicates the protein species of expected molecular weight. **Supplemental Figure S3**. **Multiple sequence alignment of *N. fowleri* Gα subunits.** The 13 candidate *N. fowleri* Gα subunits and the sole Gα subunit of *E. histolytica* are aligned using T-coffee. Coloration reflects percent sequence identity. The three switch regions, phosphate binding loop (P-loop), and nucleotide-interacting NKxD motifs are highlighted. **Supplemental Figure S4**. The RGS domain of Nf 7TM RGS2 directly binds Nf Gα2 and Nf Gα3. Surface plasmon resonance was utilized to quantify direct binding of the RGS domain from Nf 7TM RGS2 with selected Gα subunits in three nucleotide states. By equilibrium binding analysis, affinities for Nf Gα2 and Nf Gα3 in AMF-bound states were 630 ± 190 nM and 550 ± 160 nM, respectively. Kinetics of association and dissociation are also quantified. **Supplemental Figure S5**. **Nf 7TM RGS4 directly binds Nf Gα3.** Surface plasmon resonance was utilized to quantify direct binding of the RGS domain from Nf 7TM RGS3 with selected Gα subunits in three nucleotide states. By equilibrium binding analysis, affinity for Nf Gα3 in AMF-bound state was 1.8 ± 0.5 μM. Non-specific interaction with Nf Gα2 was observed at high concentrations. Kinetics of association and dissociation are also quantified. **Supplemental Figure S6**. **Nf RGS-RhoGEF directly binds Nf Gα2 and Nf Gα3.** Surface plasmon resonance was utilized to quantify direct binding of the RGS domain from Nf RGS-RhoGEF with selected Gα subunits in three nucleotide states. By equilibrium binding analysis, affinities for Nf Gα2 and Nf Gα3 in AMF-bound states were 2.4 ± 0.5 μM and 3.3 ± 0.8 μM, respectively. Kinetics of association and dissociation are also quantified.
