# Supplementary material for: Self-activating G protein α subunits engage seven-transmembrane regulator of G protein signaling (RGS) proteins and a Rho guanine nucleotide exchange factor effector in the amoeba Naegleria fowleri
Source: J Biol Chem. 2022 Jun 20;298(8):102167. doi: 10.1016/j.jbc.2022.102167 (PMC9283941; doi:10.1016/j.jbc.2022.102167)
Supplement: Supplemental Table S1 — Cloning and recombinant production of heterotrimeric G protein signaling components expressed in N. fowleri. G protein signaling components identified by open reading frame prediction and RNAseq data analysis (32) were compared with the partially annotated AmoebaDB database (amoebadb.org, (35)). The associated quantitation of relative expression, fragments per kilobase of transcript per million mapped reads (FPKM) is shown, as well as primer sequences used to attempt PCR amplification from genomic DNA. [file mmc1.pdf]

AmoebaDB accession	protein name	cloned	recombinant protein	fragment (a.a. range)	RNAseq FPKM	primer 1 (5' -> 3')	primer 2 (5' -> 3')
NF0104800*	Nf Gα1	yes	yes	11-343	6.5	GAAATAAAACGCCATATC	CTAAATAATGTTCAAATC
NF0010320*	Nf Gα2	yes	yes	17-352	6.2	GAGAGAGAAATTAAAGTG	TTAGAACAGACCTTGTTCTTTG
NF0055440	Nf Gα3	yes	yes	30-355	6.1	AGCGAAATTAAATTGTTG	TTAATCAGATCGACCAATAAC
NF0127600	Nf Gα4	failed		31-367	6.5	ATGTTCTCTTCTCTCATTT	TTACATCATGTTCAATTGC
NF0047520	Nf Gα5	yes	yes	60-394	5.5	GATGTCAAATTATTG	TTATTTATTGGAATGTTTC
NF0049200	Nf Gα6	yes	yes	11-383	5.5	GATAATACAATCAATC	TTATTTTGAAGAAAGACTC
NF0037180	Nf Gα7	yes	yes	33-353	5.7	AAGAAGATTTTGTTGGTAGG	TTAAAGATCATTCTCTGC
NF0112330*	Nf Gα8	failed		8-298	4.5**	GACTCTAAAAAGATTCTGTTA	TTATTGCTTGGACCTTGAAATA
NF0049030	Nf Gα9	no		N/A	5.5	N/A	N/A
NF0089290	Nf Gα10	no		N/A	5.9	N/A	N/A
NF0046820	Nf Gα11	no		N/A	5.9	N/A	N/A
NF0047740	Nf Gα12	no		N/A	5.9	N/A	N/A
NF0049340	Nf Gα13	no		N/A	5.7	N/A	N/A
NF0001660	Nf Gβ1	yes	no	1-355 (full)	5.8	GATGCCGCTCCCATCAC	TTAGGCCCAGATTTGCAA
NF0108330	Nf Gβ2	no		N/A	5.5	N/A	N/A
N/A	Nf Gγ1	yes	no	1-78 (full)	N/A	ATGAATAAAATGGCAAAC	TTACATGATGGTACAACA
NF0059410	NfGPCR1	no		N/A	5.9	N/A	N/A
NF0014590*	Nf 7TM RGS1	yes	yes	1036-1215	6	AACAAAGACATGAGTATC	TTAACACAAGTACACCACATTC
NF0023670*	Nf 7TM RGS2	yes	yes	1262-1419	5.6	AATCAAACGGAATTTCAA	TTACAAAAGATTTACTTG
NF0083860*	Nf 7TM RGS3	yes	yes	1539-1678	5.1**	ACTGAACTTCAAG	TTACAAAAGATTTACTTG
NF0094010*	Nf 7TM RGS4	yes	yes	2293-2459	5.4	AATAAGGAAATGC	CTAAATGCCGTACACCAC
NF0047690	Nf RGS-RhoGEF	yes	yes	590-719	5.5	TCGGAAAATCTTGTGTCTG	TGTTTTCATTTGCTCATC
NF0029190	Nf PLC1	yes	no	291-720	5.3	ATGATGAGAAGACCAGAA	TCACATGAATTCATGATG
NF0108600	Nf PLC2	yes	no	175-630	5.5	ATGGACGCAAAAATTATGG	TGAACAGTGTTCAATGAC
NF0057440	Nf PLC3	no		N/A	5.5	N/A	N/A
NF0009590	Nf AC1	yes	no	1474-1667	5.2	GAAGAGAAAGCCAAG	TTCTTCAAATTCCAAAC
NF0046870	Nf AC2	failed		1490-1683	5.2	GAAGAGAAAGCCAAG	TTCTTCAAATTCCAAAC
NF0022680	Nf AC3	failed		555-747	5.3	GAAGAGAAAAAGAAGAGTG	CTTTTCAAATCGATATTTTCC
NF0051780	Nf AC4	failed		333-504	5.5	GAGGAAAAACAACG	CTCACGCTCTTCGAAATC
NF0001350	Nf AC5	yes	no	500-705	5.4	ACTGCTGTCATG	CCTTCTCAAAACAAATC
NF0074420	Nf AC6	yes	no	1425-1618	5.3	GAAGAGAAAGCCAAATC	TTCTTCAAATTCCAAAC
NF0020430	Nf Arrestin1	yes	no	94-274	5.3	ATGAAATATTTCATTG	TTACTTCCAGCAACCCT
NF0068480	Nf Arrestin2	yes	no	1-466	5.4	ATGGACAAAAACACG	TTAATCCTTCTTTGG
NF0046300	Nf Arrestin3	yes	no	155-521	5.7	ATGCCAGCCATTGACTATG	TTAGTAACTTGGATAATTCACTGC

* partial sequence in AmoebaDB

** RNAseq FPKM expression level below 20th percentile among all transcripts
