# Supplementary material for: Self-activating G protein α subunits engage seven-transmembrane regulator of G protein signaling (RGS) proteins and a Rho guanine nucleotide exchange factor effector in the amoeba Naegleria fowleri
Source: J Biol Chem. 2022 Jun 20;298(8):102167. doi: 10.1016/j.jbc.2022.102167 (PMC9283941; doi:10.1016/j.jbc.2022.102167)
Supplement: Supplemental Figure S1 — Phylogenetic analysis of N. fowleri Gα subunits. Phylogenetic analysis of Gα subunits from N. fowleri, humans, and select model organisms was performed with bootstrapping based on a multiple sequence alignment. Branch lengths are represented to scale, and branch points are labeled with bootstrap values. Supplemental Figure S2. Isolation of recombinant N. fowleri proteins produced in E. coli. A Coomassie brilliant blue stained gel demonstrates enriched recombinant proteins achieved by a single nickel-NTA affinity step and gel filtration chromatography. ∗ indicates the protein species of expected molecular weight. Supplemental Figure S3. Multiple sequence alignment of N. fowleri Gα subunits. The 13 candidate N. fowleri Gα subunits and the sole Gα subunit of E. histolytica are aligned using T-coffee. Coloration reflects percent sequence identity. The three switch regions, phosphate binding loop (P-loop), and nucleotide-interacting NKxD motifs are highlighted. Supplemental Figure S4. The RGS domain of Nf 7TM RGS2 directly binds Nf Gα2 and Nf Gα3. Surface plasmon resonance was utilized to quantify direct binding of the RGS domain from Nf 7TM RGS2 with selected Gα subunits in three nucleotide states. By equilibrium binding analysis, affinities for Nf Gα2 and Nf Gα3 in AMF-bound states were 630 ± 190 nM and 550 ± 160 nM, respectively. Kinetics of association and dissociation are also quantified. Supplemental Figure S5. Nf 7TM RGS4 directly binds Nf Gα3. Surface plasmon resonance was utilized to quantify direct binding of the RGS domain from Nf 7TM RGS3 with selected Gα subunits in three nucleotide states. By equilibrium binding analysis, affinity for Nf Gα3 in AMF-bound state was 1.8 ± 0.5 μM. Non-specific interaction with Nf Gα2 was observed at high concentrations. Kinetics of association and dissociation are also quantified. Supplemental Figure S6. Nf RGS-RhoGEF directly binds Nf Gα2 and Nf Gα3. Surface plasmon resonance was utilized to quantify direct binding of the RGS [file mmc2.pdf]

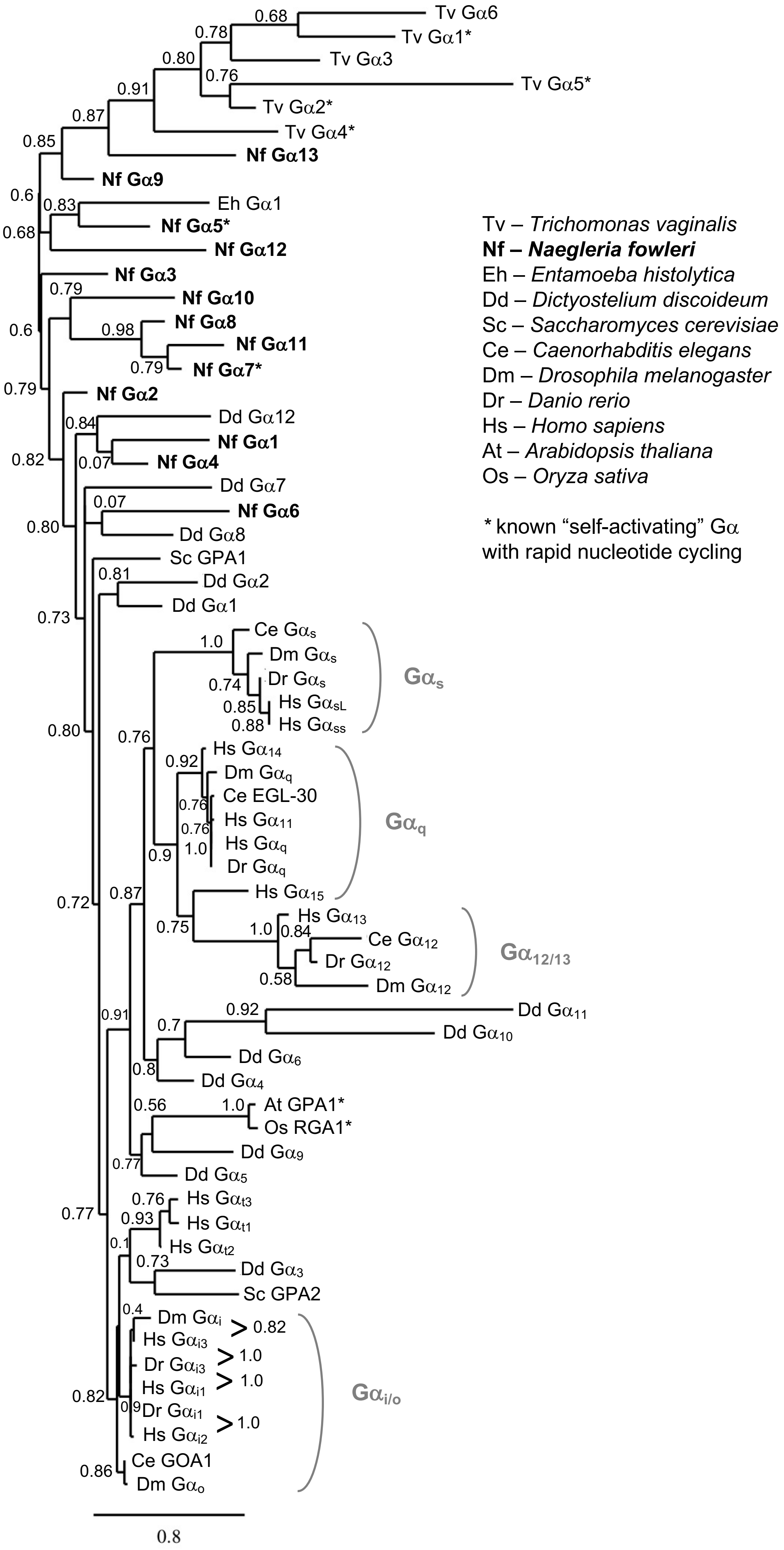
Tv Gα6
Tv Gα1*
Tv Gα3
Tv Gα5*
Tv Gα2*
Tv Gα4*
Nf Gα13
Nf Gα9
Eh Gα1
Nf Gα5*
Nf Gα12
Nf Gα3
Nf Gα10
Nf Gα8
Nf Gα11
Nf Gα7*
Nf Gα2
Dd Gα12
Nf Gα1
Nf Gα4
Dd Gα7
Nf Gα6
Dd Gα8
Sc GPA1
Dd Gα2
Dd Gα1
Ce Gαs
Dm Gαs
Dr Gαs
Hs GαsL
Hs Gαss
Gαs
Hs Gα14
Dm Gαq
Ce EGL-30
Hs Gα11
Hs Gαq
Dr Gαq
Gαq
Hs Gα15
Hs Gα13
Ce Gα12
Dr Gα12
Dm Gα12
Gα12/13
Dd Gα11
Dd Gα10
Dd Gα6
Dd Gα4
At GPA1*
Os RGA1*
Dd Gα9
Dd Gα5
Hs Gαt3
Hs Gαt1
Hs Gαt2
Dd Gα3
Sc GPA2
Dm Gαi
Hs Gαi3
Dr Gαi3
Hs Gαi1
Dr Gαi1
Hs Gαi2
Ce GOA1
Dm Gαo
Gαi/o
0.8
Tv – Trichomonas vaginalis
Nf – Naegleria fowleri
Eh – Entamoeba histolytica
Dd – Dictyostelium discoideum
Sc – Saccharomyces cerevisiae
Ce – Caenorhabditis elegans
Dm – Drosophila melanogaster
Dr – Danio rerio
Hs – Homo sapiens
At – Arabidopsis thaliana
Os – Oryza sativa
* known "self-activating" Gα with rapid nucleotide cycling

Figure S1

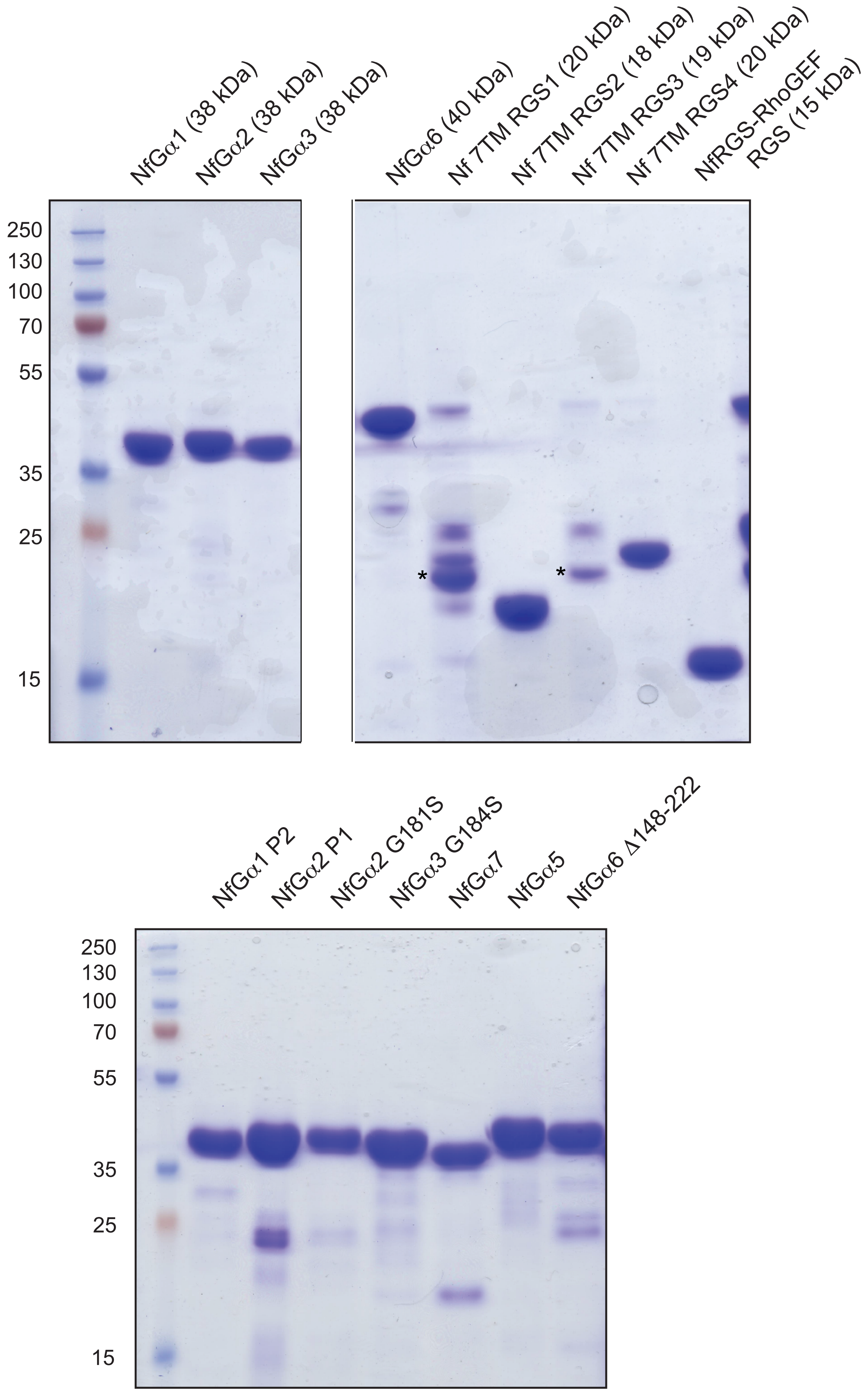
NfGα1 (38 kDa)
NfGα2 (38 kDa)
NfGα3 (38 kDa)
NfGα6 (40 kDa)
Nf 7TM RGS1 (20 kDa)
Nf 7TM RGS2 (18 kDa)
Nf 7TM RGS3 (19 kDa)
Nf 7TM RGS4 (20 kDa)
NfRGS-RhoGEF RGS (15 kDa)
250
130
100
70
55
35
25
15
*
*
NfGα1 P2
NfGα2 P1
NfGα2 G181S
NfGα3 G184S
NfGα7
NfGα5
NfGα6 Δ148-222
250
130
100
70
55
35
25
15

Figure S2

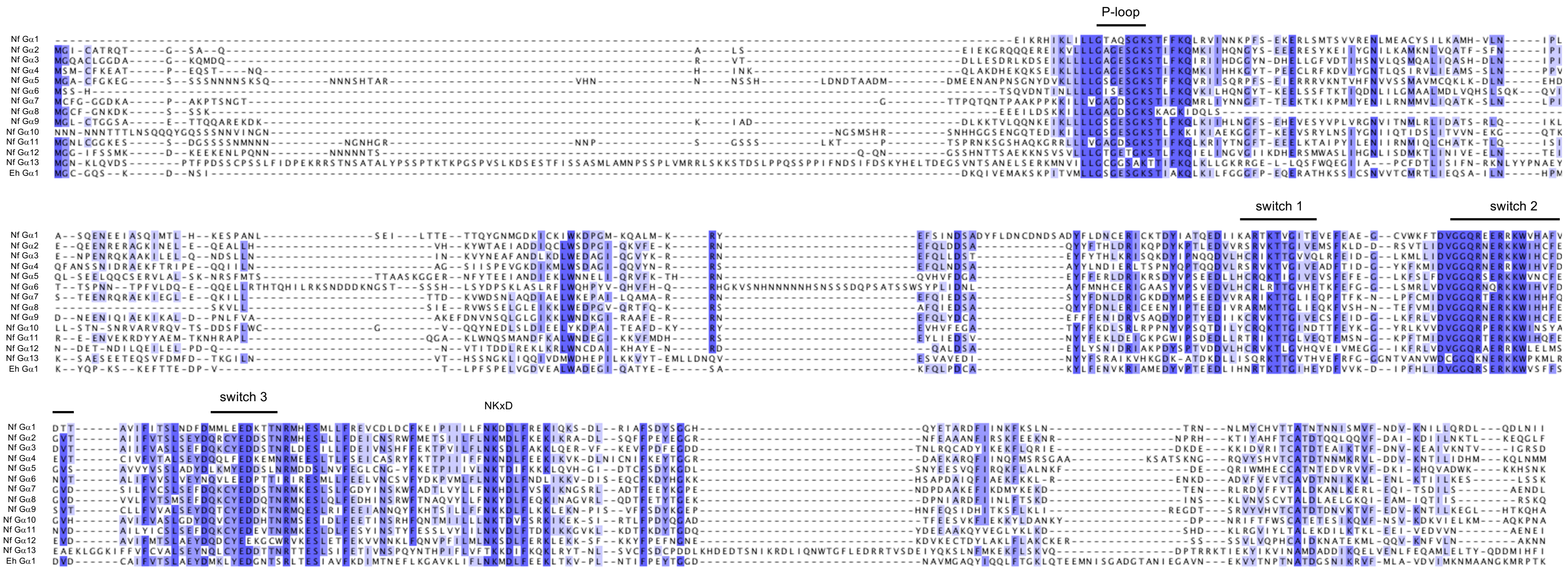
P-loop
switch 1
switch 2
switch 3
NKxD
Nf Gα1
Nf Gα2
Nf Gα3
Nf Gα4
Nf Gα5
Nf Gα6
Nf Gα7
Nf Gα8
Nf Gα9
Nf Gα10
Nf Gα11
Nf Gα12
Nf Gα13
Eh Gα1

Figure S3

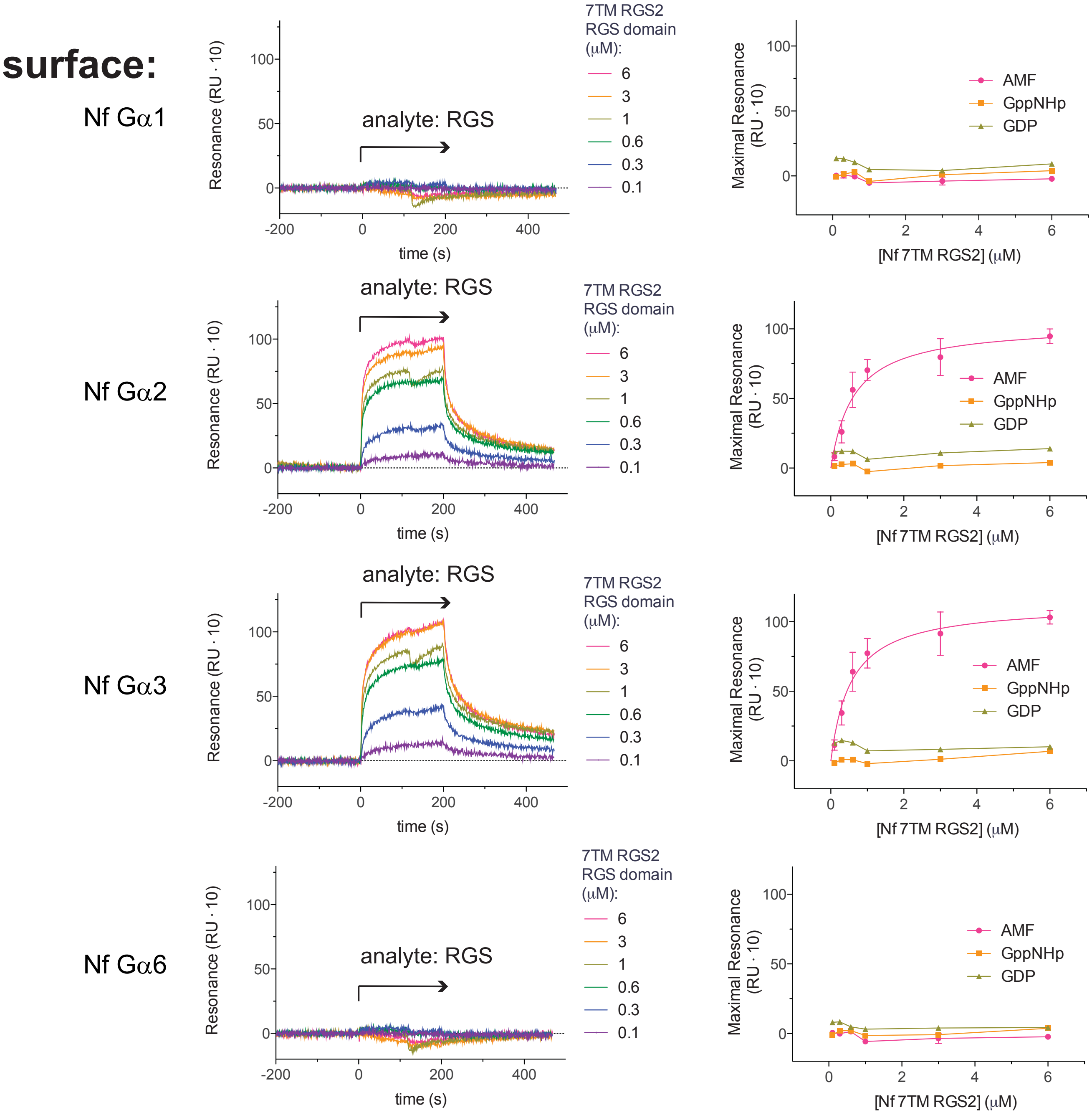


| | $k_a$ ($\mu M^{-1}s^{-1}$) | $k_d$ ($s^{-1}$) | $K_D$ (nM) |
|---|---|---|---|
| Nf Gα2 | 0.145 +/- 0.007 | 0.045 +/- 0.001 | 610 |
| Nf Gα3 | 0.122 +/- 0.006 | 0.016 +/- 0.003 | 220 |

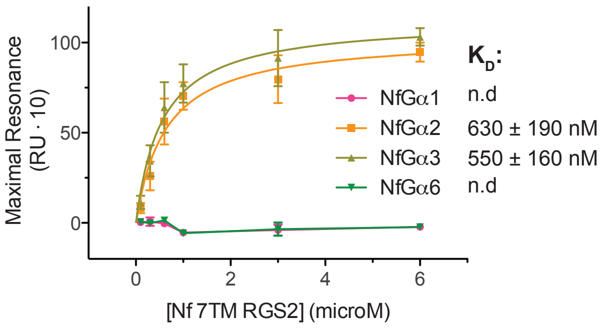


Figure S4

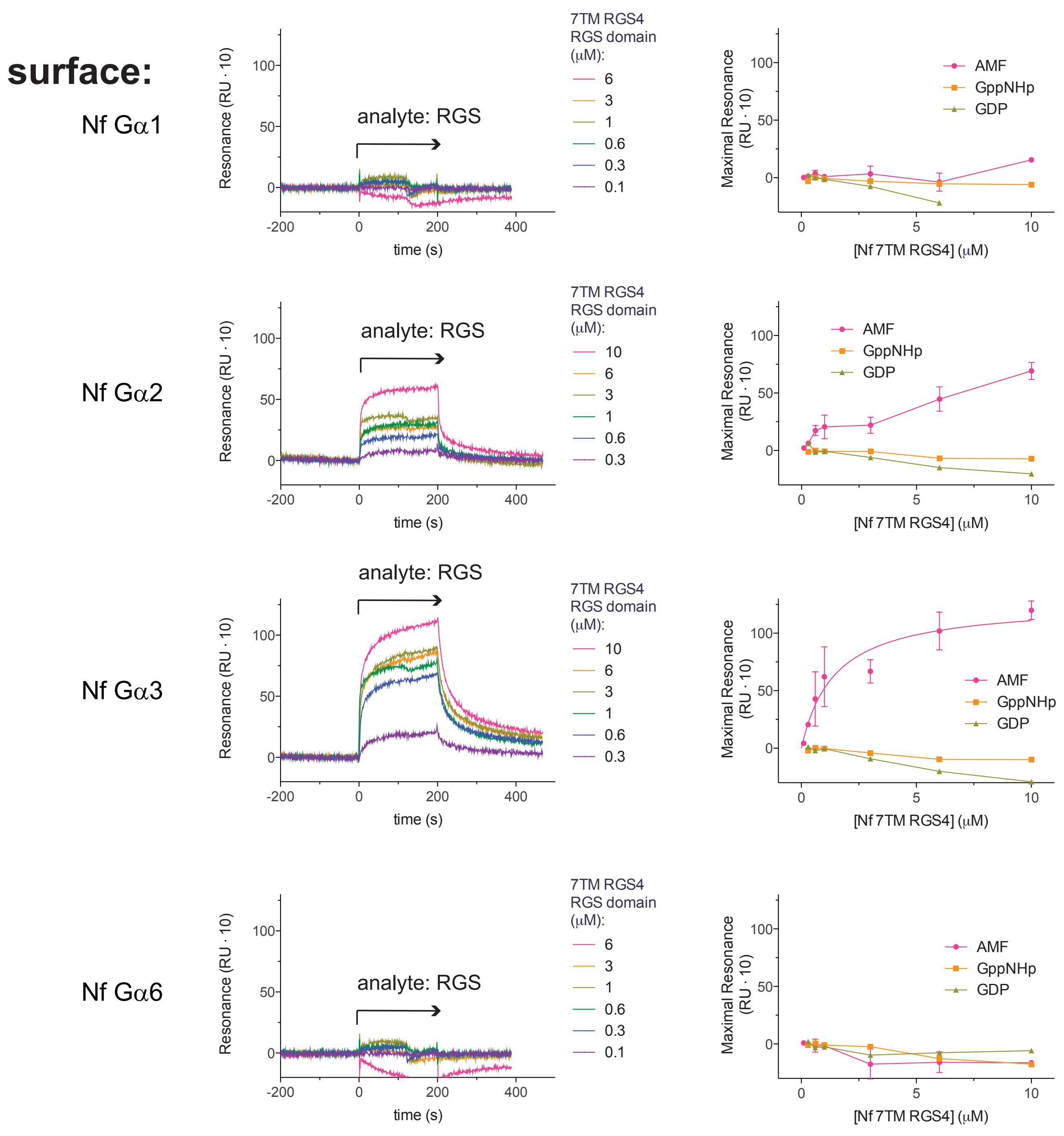


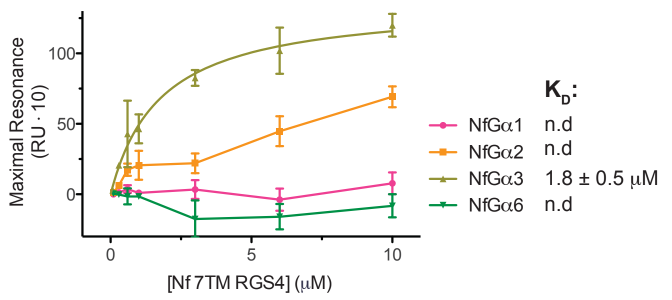


| | $k_a$ ($mM^{-1}s^{-1}$) | $k_d$ ($s^{-1}$) | $K_D$ (μM) |
|---|---|---|---|
| Nf Gα3 | 0.184 +/- 0.005 | 0.018 +/- 0.003 | 3.0 |

Figure S5

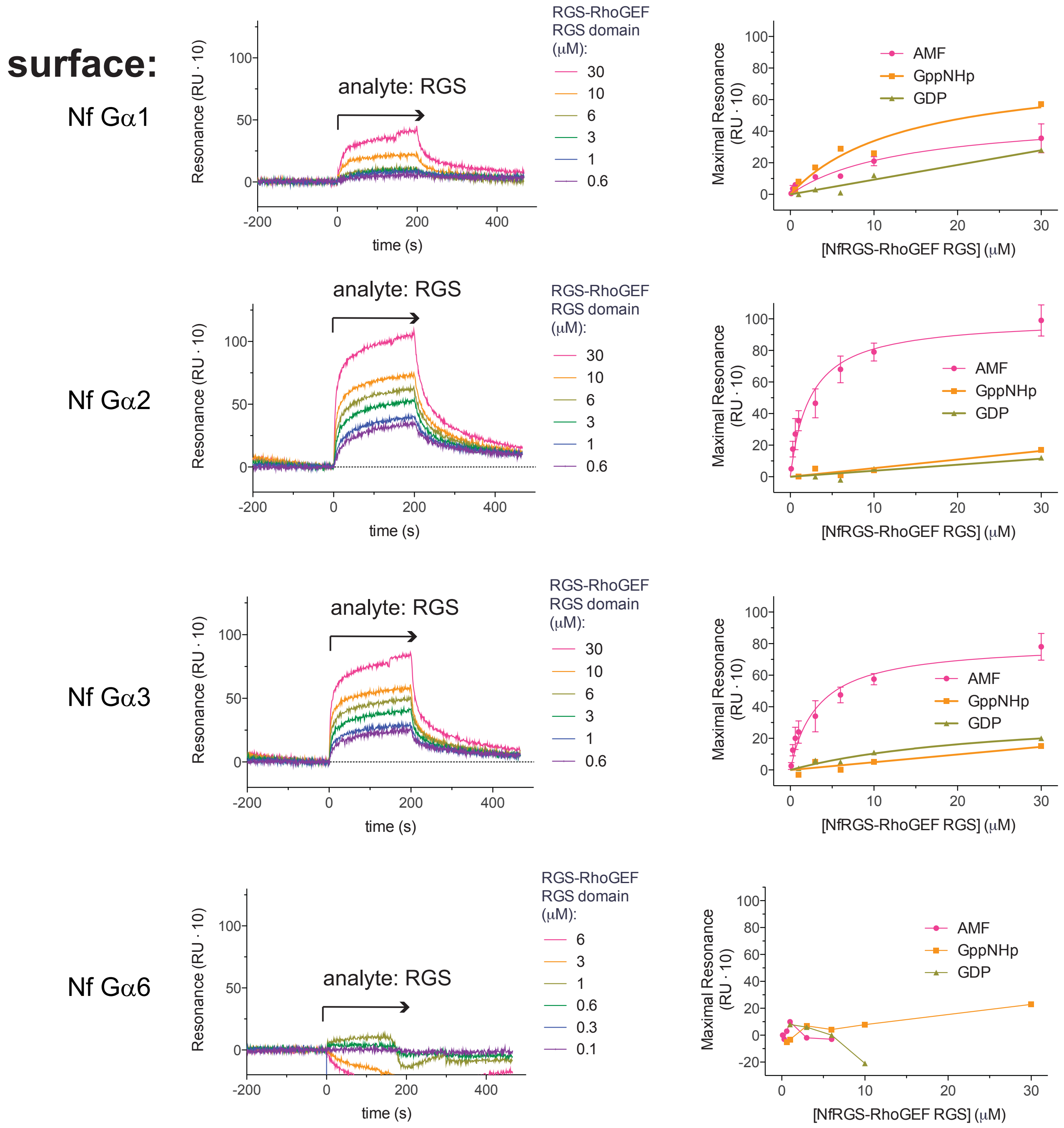


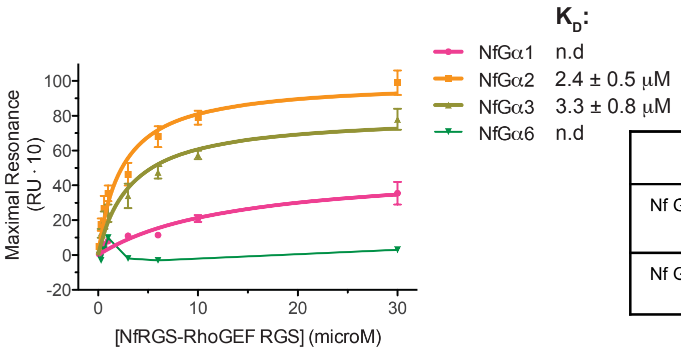


| | $k_a$ ($mM^{-1}s^{-1}$) | $k_d$ ($s^{-1}$) | $K_D$ (μM) |
|---|---|---|---|
| Nf Gα2 | 5.2 +/- 0.1 | 0.0072 +/- 0.0001 | 1.4 |
| Nf Gα3 | 7.1 +/- 0.1 | 0.0110 +/- 0.0001 | 1.6 |

Figure S6
